# Clinico-morphological patterns of breast cancer including family history in a New Delhi hospital, India-A cross-sectional study

**DOI:** 10.1186/1477-7819-3-67

**Published:** 2005-10-13

**Authors:** Sunita Saxena, Bharat Rekhi, Anju Bansal, Ashok Bagga, Chintamani, Nandagudi S Murthy

**Affiliations:** 1 Institute Of Pathology-ICMR, Safdarjung Hospital Campus, New Delhi – 110029. India; 2 Department of Surgery, Safdarjung Hospital, New Delhi – 110029. India; 3 Emeritus Scientist (Statistics), Indian Council of Medical Research, New Delhi – 110029. India

## Abstract

**Background:**

Breast cancer is the second most common malignancy among women, next to cervix cancer. Understanding its pathogenesis, morphological features and various risk-factors, including family history holds a great promise for the treatment, early detection and prevention of this cancer.

**Patients and methods:**

In an attempt to evaluate the clinico-morphological patterns of breast cancer patients, including their family history of breast and/or other cancers, a detailed analysis of 569 breast cancer cases diagnosed during the years 1989–2003 was carried out. Mean and standard deviation and Odds ratios along with 95% confidence intervals were estimated. χ^2^/Fisher's exact test were employed to test for proportions.

**Results:**

Mean age of the patient at presentation was 47.8 years, ranging from 13–82 years. Among the various histo-morphological types, Infiltrating duct carcinoma (IDC) was found to be commonest type i.e. in 502 cases (88.2%), followed by infiltrating lobular carcinoma (ILC) in 21 cases (3.7%) and other types forming 9(1%). Out of 369 cases where TNM staging was available, stage IIIB (35.2%) was the commonest. Lymph node positivity was observed in 296 cases (80.2%). Out of 226 cases evaluated for presence of family history, 47 cases (20.7%) revealed positive family history of cancer, among which breast or ovarian cancer were the commonest type (72.0%). Patients below 45 years of age had more frequent occurrence of family history as compared to above 45 years. Amongst familial cases, Infiltrating duct carcinoma was the commonest form accounting for 68.8% cases while ILC was found to be in a higher proportion (12.5%) as compared to non- familial cases (5.4%).

**Conclusion:**

Among the various determining factors for development of breast cancer and for its early detection, family history of cancer forms one of the major risk factor. It is important to take an appropriate history for eliciting information pertaining to occurrence of cancers amongst the patients' relatives there by identifying the high risk group. Educating the population about the risk factors would be helpful in early detection of breast cancer.

## Background

Breast Cancer is the most frequent cancer in women worldwide with 1.05 million new cases every year and represents over 20% of all malignancies among females [1]. Over 50% of breast cancer incidence occurs in the developed world. High-risk areas include Europe and North America. The lowest rates are reported from Africa and Asia. However, it still ranks as the commonest cancer among women in these regions. Incidence of breast cancer is increasing in most of the countries, including the areas, which have had previously low rates [2-4]. It is estimated that in 2001 there were approximately 80,000 new breast cancer cases in India [5]. The population based cancer registry data from the various parts of the country, has revealed breast cancer as the commonest cancer among women in Delhi, Mumbai, Ahmedabad, Calcutta and Trivandrum. In the rest of the other Indian registries, breast cancer is listed as the second leading site among women [6,7]. The age standardized incidence rates vary between 9–32 per 100,000 women. An increasing trend in the incidence rates of the breast cancer has been reported from the various registries of National Cancer Registry Project [7]. This malignancy accounts for 19–34% of all cancer cases among women nationally. While the epidemiological studies for breast cancer carried out in India have largely focused on risk factors such as, religion, age at menarche, menopause and reproductive history, not much attention has been paid on role of family history even though genetic predisposition is responsible for 5–10% of all breast cancers [8-17]. It is well known that the hereditary factors play a far greater role in women for the development of breast cancer [18-20]. Data so far available is from the western studies. Several clinical and morphological parameters such as histological type of tumor, tumor grade, axillary lymph node involvement, bilaterality etc. have been established as the predictors of tumor behavior in breast cancer patients. These prognostic factors are indicators of the inherent aggressiveness of the tumor as well as of the extent of the disease and based on these factors, treatment decisions are being taken up by the clinicians. The association of these prognostic factors with familial cancers has not been documented.

The present study attempts to describe some of the clinico-pathological features of the breast cancer cases seen at a tertiary level hospital in the city of Delhi. The data has been analyzed by various parameters such as age, religious groups, morphological patterns of tumor, lymph node status, TNM staging and a significant parameter lately included i.e. familial history.

## Patients and methods

A total of 788 specimens in the form of cytology and biopsy, which were clinically diagnosed as breast cancer cases were received at Institute of Pathology (IOP) during the period 1989–2003. The following eligibility criteria were employed for inclusion of cases into the study, (a) patients with complete clinical details, containing the personal proforma (b) patients who were residents of India and (c) histologically/cytologically confirmed cases of breast cancer.

Of the 788 cases, 569 cases (72.2%) satisfied the eligibility criteria and were included for the present analysis. The detailed breakup regarding 218 ineligible cases was as follows: 104 were benign lesions on histopathological examination and 40 were inflammatory lesions. Eight cases were of cystosarcoma phylloides. In 42 cases, the tissue submitted was inadequate and in the rest 25 cases clinical history was not available. For all those cases, which satisfied the eligibility criteria, clinical history and brief information on socio demographic parameters viz. age, religion, marital status and parity were extracted from the clinical records. Information relating to bilaterality of the cancer was recorded after the pathological diagnosis. Data on family history of cancers were recorded up to second degree after an in depth interview with the patient or with the person accompanying the patient. A thorough rapport was established before collecting the information regarding the family history. Wherever possible, patients were requested to bring the diagnostic reports relating to their family members to confirm the authenticity of family history of cancer. Collection of information relating to the family history was started only after 1998. The clinical extent of the disease at presentation was assessed according to the UICC TNM classification [21]. The religion wise distribution of the population for the city of Delhi was obtained from the reports of the latest census [22].

### Clinical breast and axillary lymph node examination

In clinically selected cases, triple approach including clinical examination coupled with ultrasonographic examination (USG) and mammography was also carried out. USG was carried out to assess the extent of the axillary lymph nodes more precisely. Axillary lymph node examination was done clinically as well as by ultrasound. Most of the patients of breast cancer that we receive are locally advanced cancers and have palpable lymph nodes. As a matter of policy, axillary clearance was thus done in most patients. There was also a small percentage of patients who did not have palpable lymph nodes,- for them axillary lymph node sampling was done and if the level 1 lymph nodes were found to be positive, further management was done.

### Statistical analysis

The data were tabulated and analyzed. Mean and standard deviation (SD) were computed for quantitative data. The statistical significance of associations between the various qualitative parameters was evaluated through Chi-square (χ^2^) test/Fisher's exact test (two tail). In view of skewness in the age data the statistical significance of means was tested through the non-parametric Mann Whitney test (in case of two groups) or Kruscal Wallis one- way analysis of variance for more than two groups. P value ≤ 0.05 was considered statistically significant (S). Odds ratio (OR) along with 95% confidence intervals (95% CI) were also estimated, wherever applicable.

## Results

### Age

In the present series the average age of the patient at presentation was 47.8 years with a standard deviation of 12.2 years. The youngest patient was 13 years and oldest patient was 86 years old. The commonest age group of incidence was 45–54 years (31.8%). Nearly 22% of cases were below 40 years while 16% of cases were above the age of 65 years.

### Religion

Analysis of data by major religious groups indicated that 87%, 7.7% and 5.3% belonged to Hindus, Muslims and Christian communities respectively. It may be noted that while Christian population constituted only 0.88% of the total population in the city of Delhi as per the reports of latest census of 2001 the cancer cases in this community accounted for 5.3%. The mean age at presentation according to various religions was found to be 48.1 (SD = 12.28), 42.4 (SD = 9.15) and 50.8 (SD = 12.59) years for Hindu, Muslim and Christians respectively. Christians had higher age at presentation. The differences in the mean age at presentation among different groups were found to be statistically significant (P < 0.05).

### Histo-morphological types

In the present study, various morphological variants and patterns were observed. The histomorphological types seen among 569 female breast cancers indicated that there were 502 cases (88.2%) with histology of IDC not otherwise specified (NOS), which was found to be the most common type. This was followed in decreasing order by infiltrating lobular carcinoma in 21 cases (3.7%); colloid carcinoma in 6 cases (1.1%), ductal carcinoma-in-situ in 6 cases (1.1%), metaplastic type in 5 cases (0.9%), schirrous carcinoma in 5 cases (0.9%), apocrine type in 4 cases (0.7%) and the rest 20 cases (3.5%) with other types of carcinoma (Table 1).

**Table 1:** Distribution of cases according to histomorphological types

**Histomorphological type**	**No.**	**%**
Infiltrating duct carcinoma. [IDC]	502	88.2
Infiltrating lobular carcinoma. [ILC]	21	3.7
Colloid carcinoma.	6	1.1
Ductal carcinoma-in-situ	6	1.1
Metaplastic carcinoma.	5	0.9
Schirrous carcinoma.	5	0.9
Apocrine carcinoma.	4	0.7
Medullary carcinoma.	6	1.1
Tubular carcinoma.	3	0.5
Cribriform carcinoma.	3	0.5
Mixed ductal & lobular carcinoma.	5	0.9
Other carcinomas	3	0.5
Total	569	100

Others consisted of Epidermoid, Papillary and juvenile Secretory carcinomas

### Reproductive history

Out of 569 cases of breast cancer, it was observed that 6 cases (1.1%) were unmarried. Out of these, 3 were in the age group 20–29 years and remaining 3 cases were in the age group 30–39 years. Amongst the 563 married women, four cases (0.7%) revealed a history of nulliparity. Out of these 4 cases, 3 cases were in 40–49 years and remaining one case was in the age group 50–59 years old.

### Bilaterality

It was noted that out of 569 cases, 564 (99.1%) cases presented with unilateral breast lump that was proven to be cancerous. Five cases presented with bilateral breast lumps, subsequently proven to be cancerous. Out of these, 2 were synchronous and 3 were metachronous. Among the 5 bilateral cases, 3 cases revealed a positive family history of cancer.

### TNM staging

Of the 569 cases, TNM staging was available for 369 patients. The most commonly observed stage of presentation was IIIB with 130 cases (35.2%) followed in decreasing order of frequency by stages IIIA with 100 cases (27.1%), IIB with 60 (16.3%) cases, IV with 29 cases (7.9%) and stage I with only 5 cases (1.4%) (Table 2). Of the 29 metastatic cases, 6 cases had supraclavicular lymph nodes, 18 had hepatic and 5 cases had both hepatic and skin deposits. The mean age of cases at presentation was found to be 45.0 years (SD = 14.15) for cases in stage IIA as compared to 51.1(SD = 13.67) years for cases in stage IV. The mean age at presentation showed an increasing trend for the various stages, viz – stage IIB (48.4 years, SD = 10.69), IIIA (49.8 years, SD = 10.76), IIIB (50.6 years, SD = 11.6) and was found to be statistically significant (p < 0.05).

**Table 2:** Distribution of cases by stage, lymph node status and mean age.

**Characteristics**	**TNM grading**	**No.**	**Percentage**	**Mean Age (SD)**
*TNM stage [N = 369]*				
Stage I	T1N0M	5	1.4	N.C
Stage IIA		45	12.2	45.0(14.15)
	T0N1M0	2		
	T1N1M0	5		
	T2N0M0	38		
Stage IIB		60	16.3	48.4(10.69)
	T2N1M0	37		
	T3N0M0	23		
Stage IIIA		100	27.1	49.8(10.76)
	T2N2M0	1		
	T3N1M0	67		
	T3N2M0	32		
Stage IIIB		130	35.2	50.6(11.8)
	T4 any NM0	126		
	Any T N3M0	4		
Stage IV	Any T any NM1	29	7.9	51.1(13.67)
*Lymphnode status (N = 369)*				
Present		296	80.2	48.5(11.71)
Absent		73	19.8	45.8(13.43)

Percentages are against the total number of cases.

NC-Mean age (years) was not calculated due to small number of cases.

### Lymph node status

Lymph node positivity was analysed histologically in all the 369 cases. Histopathological confirmation of lymph node involvement pN was observed in 296 cases (80.2%) (Table 2). The mean age of cases with lymph node positivity was found to be 48.5 (11.7) years as compared to 45.8 (SD = 13.43) years in negative cases. However, the differences were found to be statistically not significant

### Association of religion with histo-pathological type, TNM staging and lymph node status

An analysis was carried out to understand, if any relationship existed between the religious affiliation of patient with *histo-pathological type*, TNM staging and lymph node status as the cultural patterns are different in the three religions (Table 3). The three histo-pathological types of disease viz. IDC, ILC and "all other tumors combined together" were considered for the above analysis. Percentage patients with these histopathological types were found to be similar in all the three religious groups (p = 0.66). Percentage patients presenting with localized tumors (TNM stage I & II) were found to be slightly higher amongst Christians (39.1%) as compared to other two religions (29.6% and 24.1%). However, the differences were not found to be statistically significant amongst the three religious groups (p = 0.84). Similarly, even the lymph node involvement was also observed to be almost similar in all the religious groups (p = 0.38).

**Table 3:** Association of religion with histological type, TNM stage and lymph node status [Percentage distribution]

**Characteristics/Religion**	**Hindu**	**Muslim**	**Christian**
*Histological type [N = 569]*	%	%	%
IDC	88.7	84.1	86.7
ILC	3.2	6.8	6.7
Others	8.0	9.1	6.7
*TNM stage [N = 369]*			
I	1.3	0.0	4.3
IIa	12.3	13.8	8.7
IIb	16.0	10.3	26.1
IIIa	27.4	24.1	26.1
IIIb	34.9	44.8	30.4
IV	8.2	6.8	4.3
*Lymphnode status [N = 369]*			
Present	79.2	79.3	91.3
Absent	20.8	20.7	8.7

Percentages are against the total number of cases seen in each religion.

### Family history

Another important epidemiological parameter included from the year 1998 was family history. The information on family history was available for 226 cases. Out of these 226 cases, 47 cases (20.7%) revealed family history of cancer. Further, stratified analysis of data, by two broad groups of less than or equal to 44 years and above 44 years revealed that, of the 93 cases below the age group of 45 years, 27 (29.0%) women gave family history of cancer in their first or/and second degree relatives. Similarly, in the other age group of above 45 years, the frequency of cancer was reported to be in 20 (15.0%) women of the 133 women belonging to above 45 years of age. The differences in the occurrence of cancer amongst the first and second-degree relatives between the above two age groups were found to be statistically significant (p < 0.05). In the families of patients below 45 years a higher incidence of cancer was noted as compared to patients above 45 years of age. All women who had family history of breast cancer in their families were considered as cases, while the rest of women were taken as controls, for estimation of odd's ratio in the age groups below and above 45 years. This was found to be 2.3 (95% CI: 1.15–4.68, p < 0.02), indicating more frequent occurrence of cancer in relatives of patients below 45 years of age. Seventy two percent of cases (n = 34) with a positive family history revealed occurrence of breast or ovarian cancers amongst their family members. Remaining 28% of patients (n = 13) with a positive family history reported other cancers in their relatives such as lymphomas, thyroid cancer, prostate and colorectal carcinomas etc. Four patients (8.5%) gave the history of multiple cancers in their family members.

Further, an attempt was made to analyze the similarity in the histo-pathological type of tumor between the familial and non-familial groups of cases. It was observed that IDC formed as the most common type of tumor in both groups of cancer (68.8% and 75.5%). However, ILC was present in a higher proportion in familial group (12.5%) as compared to non-familial cases (5.4%). Further, "all other tumors combined together" were found to be 18.7% and 19.1% respectively. The differences, was not found to be statistically significant possibly due to small number of cases observed under the familial group (p > .10)

As regards the number of family members being affected with the cancers, two patients (4.3%) revealed history of cancer in more than 4 family members amongst their first and/or second- degree relatives. Fifteen patients (31.9%) had a history of cancer in two -three family members. Further, thirty patients (63.8%) mentioned that there was only one case of cancer amongst their relatives of first and second degree. It was observed that on an average, 1.51 first and/or second degree family members had suffered from cancer in these 47 families.

## Discussion

Breast cancer incidence rates are increasing worldwide. In India, it is the most common cancer among women in many regions and has overtaken cervix cancer, which was the commonest cancer a decade ago. The continuing rise in breast cancer incidence has created an urgent need to develop strategies for prevention. Breast cancer appears to have a complex etiology, possibly with interplay of many causal factors including hormonal, genetic and environmental factors operating over a long period. Although several risk factors have been well defined, the interactions of the various etiological factors are yet to be completely understood. Moreover, analysis of newer parameters like family history could be helpful in screening high risk women for developing breast cancer, followed by planning of future, preventive and treatment modalities.

Age of the cancer patient is an important factor both for occurrence and management of the case. Average age of the patients seen in the six hospital based cancer registries in the National Cancer Registry Project (NCRP) network for the period 1994–98 was found to range from 44.2 years in Dibrugarh to 49.6 years in Bangalore and Chennai registries. In the present study the average age of the breast cancer case at presentation was found to be 47.9 years. Our findings are in agreement with the findings of the NCRP network [23]. Similarly, the average age of breast cancer patients has been reported to be 50 to 53 years in various population-based registries located in different parts of the country [7]. Similar hospital based studies carried-out at Delhi and Jaipur have also reported that the average age of breast cancer cases to be as 46.8 and 47 years [24,25]. The average age of occurrence of breast cancer amongst US white females has been reported to be 61.0 years [26]. The average age of occurrence of the breast cancer in India reveals that the disease occurs a decade earlier, as compared to western countries. The reason for early age of occurrence amongst Indian females needs to be further studied. A similar viewpoint has been put forward by a study conducted by Borovanova [27] in the Czech population. In their study, they found a shift of cancer more towards younger women.

Epidemiological studies carried out in the country have shown variation in the incidence of breast cancer among different religious groups such as Hindus, Muslims, Christians, Parsi and Buddhists. Breast cancer incidence by religion for greater Bombay population indicated highest incidence rates among Parsis and Christians and lowest rates among Jains and Buddhists. In another study from Chennai, the rates have been shown to be highest among Christians followed by Hindus and Muslims. Similarly in Trivandrum study, the incidence rates have been reported to be highest among Christians followed by Hindus and Muslims. Paymaster *et al *[14], in an analysis of hospital cancer cases by major religious groups have shown the variation in relative frequency between religious groups and in particular, high percentage among Parsi women compared to other religious communities. The reason for high incidence of breast cancer in Parsi community is as a result of their more westernized life-style, conserved genetic pool, high frequency of consanguineous marriages and higher age at the time of marriage and child birth [28]. In our study, on comparison of relative frequencies of cancer cases by various religious groups with that of population distribution of Delhi by these religious groups revealed that Christians had a higher percentage of cancer. Although Christians accounted for only 0.88% in the general population but relative frequency of cancer cases in the present study were 5.8%. The other two religions had more or less similar proportions. Although, the age at presentation amongst Christian patients was highest (51.5 years), as compared to other two religions, no significant difference was noticed in stage at presentation and lymph node status. The possible explanation for high incidence and higher age at presentation amongst Christians may be the same as in Western population.

Few studies of international variation in breast cancer have considered tumor histology. The histological type of carcinoma whether it is invasive or an *in situ *type are features which carry an inbuilt understanding of their general behavior pattern. In a study conducted by Wynder *et al *[4], comedo carcinoma and medullary carcinoma were found to be more frequent in Tokyo. Cases from a broader range of hospitals in Boston and Tokyo revealed occurrence of tumors of better prognostic types viz. intraductal, medullary and colloid types in Tokyo than in Boston. It is important to understand the relationship of histological type to etiology, and to allow separation of entities with distinct etiologies.

Histology as a prognostic factor has been well documented. Patients with histology of Infiltrating duct carcinoma (IDC)(NOS) have a poor survival compared to other types [17]. In the present study among the different histomorphological types, Infiltrating duct Carcinoma (NOS) was found to be the most common type i.e. in 502 cases (86.9%). The histological distribution of female breast cancers seen during the years 1984–93 in the hospital based cancer registries under the network of NCRP revealed that, in Mumbai, Bangalore and Thiruvananthapuram 83.3%, 85.0%, and 83.3% cases had a histology of infiltrating duct carcinoma while, 2.7%, 1.7%, 1.6%, belonged to lobular carcinoma, and the remaining 14.0%, 13.3%, 15.2% cases respectively were of other histopathological subtypes [23]. Infiltrating duct carcinoma was the commonest type seen in our series. The findings of the present study are almost in agreement with the findings reported by the hospital based cancer registries under the NCRP network. In the US population also, infiltrating ductal type of breast carcinoma was found to be the commonest histological type [29]. Among the age-correlation with various histomorphological types, prognostically better tumours like juvenile secretory were more commonly observed amongst younger females while infiltrating ductal carcinoma and lobular carcinoma remained the two most common cancers amongst elderly patients. It was also noted that ILC was present in a higher proportion in familial group (12.5%) as compared to non-familial cases (5.4%) in the present study, however it was not found statistically significant.

It is well known that breast cancer cases diagnosed at an earlier stage have a more favorable prognosis compared to those detected at late stage. However, because lack of awareness, fear of disease and psychological reasons, most of the patients in our country try to ignore or hide the disease and by the time they come to the hospital, the disease is already in the late stages. In the present study, over 90% of cases are in stages II, III and IV. The findings of the present study reveal that breast cancer frequently presents at higher stage i.e. 36.1% cases presenting with Stage IIIB in Indian population. This reflects a need for awareness and to initiate programs for early diagnosis of the cancer.

Numerous studies carried out in India and western populations have identified various reproductive factors generally associated with breast cancer [9-11,13,29-31]. A case-control study to identify risk factors for breast cancer carried out in Mumbai, India indicated that single women compared to married women had 4–5 fold higher risk for development of breast cancer in the age group of 40–54 years and 55 and above [14]. In another study it has been shown that nulliparous women had 2.2-fold higher risk than parous women [16]. High incidence of breast cancer among Parsi women was partly due to more unmarried women, late age at marriage and first child, less children and consanguinity of marriage [28]. Nulliparity and late age at first birth are the consistently observed reproductive risk factors [32]. In our study only 6 cases (1.0%) were found to be unmarried and four married woman (0.6%) were nulliparous.

Family history is another risk-factor for breast carcinoma. It has been noted that women who have first degree relative with breast cancer have a risk two to three times that of general population, the risk further increased if the relative was affected at an early age and/or had bilateral disease [20]. There is a greater risk if more than one close relative is affected, if breast cancer has occurred at a young age in a family member or if a patient has bilateral disease [30]. One of the explanations for familial aspects of breast cancer is germline mutation in BRCA1, BRCA2, p53 and other genes [33]. These cellular genes, which comprise dominantly acting oncogenes and recessively acting tumour suppressor genes, have been shown to contribute to genetic predisposition to variety of human cancers. In the present study, 20.2% cases revealed a positive family history of cancer. Out of these, higher percentages of cases 29.0% were observed in females under 44 years of age as compared to 15% in women of above 45 years of age. The presence of family history doubled the risk of subsequent breast cancer among younger women. In a similar study by Marcus *et al*, the relative risk of breast cancer was found to be doubled by the presence of a family history of breast cancer and amplified by younger age. It has been stated that there might be considerable underestimation of hereditary breast cancers. The studies carried-out at Jaipur and Delhi have reported the family history of cancer as 10% and 8% in their series. With documentation of pedigree, familial breast cancer (FBCs) may constitute as high as one third of the total incidence of breast cancers and approximately one forth of them would fall into the subset of hereditary breast cancer (HBC). It has been observed that familial breast cancer patients have an improved rate of survival, thereby indicating importance of noting familial cancer cases [33]. The present study clearly implies the importance of taking an appropriate history for eliciting family histories from the relatives. It is crucial to elicit detailed personal and family history extending back to atleast three generations, checking the medical records including pathology reports, wherever possible it is better to complete an accurate pedigree and taking family history from both maternal and paternal side of family. History of cancers among family members helps in identifying high risk groups, who can be counselled and subjected to careful follow ups with early diagnostic modalities and can even choose certain therapies. Improved ways of follow up with study of various interacting genes would also be useful to identify high-risk groups. Therefore, it is imperative to include parameters like family history for better understanding of breast cancer causation and predisposition.

Breast cancer usually presents with a single hard lump as is evident in the present study with occurrence of bilaterality in only 5 out of total 569 cases. In an earlier study, bilaterality was reported in 3% of breast cancer patients [34]. FBC have been observed to be having improved survival, as a result of stringent monitoring which may help in diagnosis at early stage [30,35]. Clinical stage is another factor implicated. In a study conducted by Langlands *et al *[35], FBC cases had a lower stage of presentation. However, in our study, no such association was observed.

## Conclusion

Continuing increased incidence of breast cancer has added urgency to investigations of control and preventive measures. It is important to incorporate primary and secondary prevention measures of breast cancer as number of affected individuals is rising and the age of onset is shifting towards younger age groups. Efforts should be made to detect breast cancer at the very early stage through periodic screening of high-risk groups either by physical self-examination or by self-breast examination. Mammography will be difficult to implement in Indian situation for the control of breast cancer. Efforts should be made to elicit information related to factors like family history of breast cancer, thereby identifying high-risk groups. There is a need to educate both high risk and all women about the importance of Breast self Education (BSE) through press and electronic media, thus more cases can be diagnosed at an early stage and effective treatment can be given to those women and their lives can be saved.

## Competing interests

The author(s) declare that they have no competing interests.

## Authors' contributions

**SS**, The study was carried out under the overall supervision and guidance, have given final approval of the version.

**BR **Involved in the diagnostic and analytical part of the study, alongwith preparation of the manuscript

**AB**, Involved in the diagnostic and analytical part of the study

**As B**, Collection of data

**C**, Collection of clinical details & preparation of manuscript

**NSM**, Design, analysis and interpretation of data and preparation of the manuscript

## Funding

None declared
